# Expression of the IAP protein family acts cooperatively to predict prognosis in human bladder cancer patients

**DOI:** 10.3892/ol.2013.1150

**Published:** 2013-01-23

**Authors:** XIAOCHI CHEN, TIEZHENG WANG, DEYONG YANG, JIANBO WANG, XIANCHENG LI, ZHONGZHOU HE, FENG CHEN, XIANGYU CHE, XISHUANG SONG

**Affiliations:** Department of Urology, First Affiliated Hospital of Dalian Medical University, Dalian, Liaoning 116011, P.R. China

**Keywords:** bladder cancer, inhibitors of apoptosis, cooperatively, prognosis

## Abstract

The inhibitors of apoptosis (IAPs) are a group of anti-apoptotic factors in the apoptotic pathway that render cancer cells insensitive to apoptotic stimulation. Recently, several members of the IAP family have been investigated in the context of bladder cancer, and some of these have been associated with specific clinical and pathological tumor features, and with prognosis. These data suggested that the expression of an individual nuclear IAP has an important relationship with the progression of bladder cancer. To date, there are no studies concerning the overall tendencies of IAPs and their comparative therapeutic values in bladder cancer. In this study, we investigated the overall expression trends of the five tumor-related proteins, Survivin, cIAP1, cIAP2, XIAP and Livin, in normal bladder tissues and bladder cancer tissues. We classified and compared the gene expression data of these IAPs with the corresponding clinical and pathological tumor features, and with prognosis, in the development and progression of bladder cancer. The differences in IAP expression levels between archival bladder specimens from 36 normal controls and 105 patients who underwent surgery at our facility were examined using western blot analysis. The localization and expression level of each protein in low- and high-grade bladder cancer tissues were examined through immunohistochemistry. The cytoplasmic expression levels of each protein were scored as 0 (negative), +1 (weak), +2 (medium) or +3 (strong). The nuclear expression levels of cIAP1 and Survivin were scored as 0 (0%), +1 (1–25%), +2 (26–50%) or +3 (>50%). The results demonstrated that the expression of IAPs acted cooperatively to predict prognosis in human bladder cancer patients.

## Introduction

Bladder cancer is the most common malignancy of the urinary tract system and represents an important cause of morbidity and mortality. Currently, the main therapeutic method is surgery followed by postoperative intravesical instillation. However, approximately half of non-muscle-invasive bladder cancers will relapse within five years (1), regardless of clinical prognostic variables. Therefore, an increasing number of studies have focused on the search for biomarkers that are correlated with the histopathological features and biological behavior of bladder cancer, in order to improve therapeutic strategies and predict its clinical progression.

The inhibitors of apoptosis (IAPs) are a group of anti-apoptotic factors in the apoptotic pathway that render cancer cells insensitive to apoptotic stimulation (2,3). Currently, eight IAPs have been identified in mammals, including X-linked inhibitor of apoptosis (XIAP), cellular IAP1 (cIAP1), cIAP2, Livin, IAP-like protein 2 (ILP2), neuronal apoptosis inhibitory protein (NAIP), Survivin and BRUCE (4–6). The IAPs are homologs with highly conserved sequences, and are structurally similar. They constitute a family of proteins that possess between one and three baculovirus IAP repeats, and several of the proteins also have a gene (RING) finger domain at the C-terminus. IAPs interact with each other through complex interactions that may lead to the inhibition of a protein or cooperative synergistic action to protect cells from apoptosis (7–9). In addition to their roles in cytoprotection, it has been demonstrated that IAPs function as broader regulators of cellular homeostasis and are involved in cell division, metabolism and the activation of multiple intracellular signaling pathways, including NF-κB, TGF-β and JNK (4).The overexpression of IAPs is highly correlated with cancer progression and resistance to chemotherapy, and is associated with a poor prognosis (10).

Recently, several members of the IAP family have been investigated in the context of bladder cancer, and some of these have been found to be correlated with specific clinical and pathological tumor features, and with prognosis. One of our previous studies demonstrated that nuclear cIAP1 expression may be strongly correlated with bladder cancer stage, grade, tumor recurrence and tumor-related mortality (11). Gazzaniga *et al* revealed that Livin may be involved in the progression of superficial bladder cancer and could be used as a marker of early recurrence (12). Li *et al* demonstrated that XIAP may be considered to be an independent prognostic marker for the early recurrence of non-muscle-invasive bladder cancer (13). Yin *et al* revealed that the Survivin nuclear labeling index (Survivin-N) is a superior biological and prognostic marker for TaT1 urothelial carcinomas of the urinary bladder (14). It is thus evident that the expression of an individual nuclear IAP has an important correlation with the progression of bladder cancer. However, the development and progression of bladder cancer is a complex process that involves a host of functional and genetic abnormalities. Moreover, IAP family members are structurally similar, and some of these are able to act cooperatively via particular pathways to regulate apoptosis and proliferation (15,16). Therefore, research into the correlation between the expression of a single IAP and the clinical and pathological parameters of bladder cancer may be limiting.

Another previous study by our research group demonstrated that the combined knockdown of Livin, XIAP and Survivin in bladder cancer cell lines could remove the barricade in the apoptotic pathway more effectively than when only a single gene was suppressed, which may suggest a potent multitargeted gene therapy for bladder cancer (17). Rodríguez-Berriguete *et al* demonstrated that the overexpression of IAPs, including XIAP, cIAP1, cIAP2, NAIP and Survivin, was involved in the development of prostate disorders (BPH, PIN and PC) (18). Lopes *et al* demonstrated that the expression of the IAP protein family was dysregulated in pancreatic cancer cells and was important for resistance to chemotherapy (19). However, prior to this investigation, there were no studies concerning the overall tendencies of IAPs and their comparative therapeutic values in bladder cancer.

In the present study, we investigated the overall expression trends of the five tumor-related proteins, Survivin, cIAP1, cIAP2, XIAP and Livin, in normal bladder tissues and bladder cancer tissues. We classified and compared the gene expression data of these IAPs with the corresponding clinical and pathological tumor features, and with prognosis, in the development and progression of bladder cancer.

## Materials and methods

### Patients and specimens

All 152 patients who were diagnosed with primary bladder transitional cell carcinoma and treated with transurethral resection of bladder tumor (TURBT) in our department from January, 2006 to December, 2007 were included in the analyses. Adequate archival tissue was available for 105 of the 152 patients. As controls, normally appearing bladder tissues were obtained from an area outside the tumor region (>1 cm) in 36 radical cystectomy patients who were not included in the 105-patient cohort. No evidence of histological changes in the normal control bladder samples was observed histopathologically. The use of the samples was approved by the Ethics Committee of Dalian Medical University, and all patients provided informed consent prior to surgery. Staff pathologists with expertise in genitourinary pathology examined all specimens. The 2002 TNM classification system was used for pathological staging, and the 2004 WHO classification system was used for pathological grading. The mean follow-up period was 41.9 months. For postoperative surveillance, cystoscopy was performed every three months for the first two years and every six months thereafter to monitor the recurrence of bladder cancer. Recurrence was defined as positive findings on cystoscopy that were confirmed by biopsy or postoperative pathological examination. Cancers detected in the ureter and/or urethra were considered second primary tumors and not local or distant recurrences.

### Antibodies

The primary antibodies used were as follows: Rabbit polyclonal antibodies against Livin (IMGENEX, San Diego, CA, USA), Survivin (Santa Cruz Biotechnology, Inc., Santa Cruz, CA, USA) and XIAP (Santa Cruz Biotechnology, Inc.); a mouse monoclonal antibody against GAPDH (KangChen Bio-tech, Shanghai, China), and goat polyclonal antibodies against cIAP1 and cIAP2 (R&D Systems, Minneapolis, MN, USA). For western blot analysis, the antibodies were diluted 1:200 (Survivin, XIAP, cIAP1 and cIAP2) or 1:1,000 (Livin and GAPDH) in TBS with 5% bovine serum albumin. For immunohistochemistry analysis, the antibodies were diluted 1:50 (Survivin, XIAP, cIAP1 and cIAP2) or 1:1,000 (Livin) in TBS.

### Evaluation of immunostaining

The slides were evaluated under a light microscope by two separate researchers (Yang and Zong) who had no knowledge of the patient outcomes. For cytoplasmic assessment, the staining intensity was scored as 0 (negative), +1 (weak), +2 (medium) or +3 (strong). For nuclear assessment, the staining intensity was calculated by averaging five randomized microscopic fields and scored as 0 (0%), +1 (1–25%), +2 (26–50%) or +3 (>50%) according to the percentage of positively stained cells in the total number of cancer cells. The combination of staining intensity for cIAP1-N and Survivin-N was scored as 0, +1–2, +3–4 or +5–6 for each sample. The combination of staining intensity for cIAP1-C, cIAP2 and XIAP was scored as 0, +1–3, +4–6 or +7–9 for each sample. The combination of staining intensity for Survivin-C and Livin was scored as 0, +1–2, +3–4, or +5–6 for each sample.

### Statistical analysis

SPSS 13.0 software (SPSS Inc., Chicago, IL, USA) was used for statistical analysis. Differences in the expression levels of the IAPs in bladder cancer cells and the normal bladder urothelium were assessed using the non-parametric Mann-Whitney U test. Associations between the expression levels for each IAP and the bladder cancer clinicopathologic features were also assessed by the non-parametric Mann-Whitney U test. The correlation between cIAP-N expression and Survivin-N expression was assessed using Spearman’s correlation analysis. Survival functions and differences were calculated by the Kaplan-Meier method and assessed using the log-rank statistic. Multivariate survival analyses were performed using the Cox proportional hazards regression model. P<0.05 was considered to indicate a statistically significant difference.

## Results

### Bladder cancer patient characteristics

A total of 105 bladder cancer patients were included in this study (75 males and 30 females), and were aged from 37–83 years (mean, 56 years). Approximately 72 patients exhibited a single tumor, and 33 patients had multiple tumors. The pathological classification system used for tumor grading considered 70 patients to be low grade and 35 patients to be high grade. There were 60 cases of non-muscle-invasive bladder cancer and 45 cases of muscle invasion. The follow-up time for the entire cohort ranged from 6–68 months (mean, 41.9 months).

### IAP expression levels in bladder cancer and normal bladder urothelium

**Figure 1:**
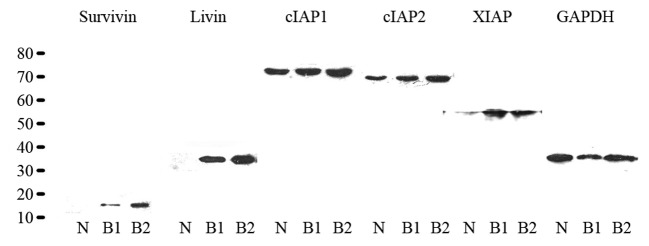
Western blot analysis of the expression of inhibitor of apoptosis (IAP) family members in normal bladder tissue and bladder cancer tissue. N, normal bladder urothelium; B1, low-grade bladder cancer; B2, high-grade bladder cancer.

Western blot analysis was performed to determine the expression levels of the IAPs in bladder cancer and normal bladder tissue. The results revealed a single band for all antibodies studied at the corresponding molecular weights in bladder cancer and normal bladder urothelium. In normal bladder urothelium, Livin and Survivin were not detected, while immunoreactivity for the other antibodies was observed at the corresponding molecular weights (Fig. 1). Differences between the expression levels of the IAP family members in bladder cancer and the normal bladder urothelium were assessed using the non-parametric Mann-Whitney U test (Table I). The results demonstrated that the IAPs were over-expressed in bladder cancer tissues compared with normal bladder tissue samples. These data were consistent with the western blot analysis results.

### Expression levels and localization of IAP family members in different pathological grades of bladder cancer tissue

**Figure 2:**
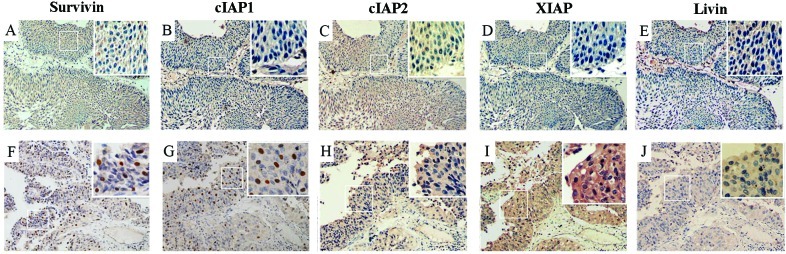
Immunohistochemical staining of inhibitors of apoptosis (IAPs) in representative sections from different grades of bladder cancer tissue. (A–E) Images from the same sample, same area, and same visual field showing serial slices of low-grade bladder cancer tissue. (F–J) Images from the same sample, same area and same visual field, demonstrating serial slices of high-grade bladder cancer tissue.

Immunohistochemistry was performed to demonstrate the presence and localization of IAP family members in different grades of bladder cancer tissue (Fig. 2). The results demonstrated that the expression levels of each IAP were increased in high-grade bladder tissue compared with low-grade tissue. cIAP1 and Survivin were observed in both the cytoplasm and nucleus, while the other IAPs were principally localized in the cytoplasm. cIAP1 and Survivin were observed to exhibit a marked tendency toward nuclear expression in the high-grade bladder cancer panel compared with the low-grade panel.

### IAP expression levels and bladder cancer features

The correlations between clinical and pathological variables, and the expression levels of each group of IAPs are shown in Table II. No significant correlation was observed between the expression of each group of IAPs and gender, age or number of tumors (P>0.05). The level of expression of each group (1, cIAP1-N+Survivin-N; 2, cIAP1-C+cIAP2+XIAP and 3, Survivin-C+Livin) was significantly associated with the presence of muscle-invasive disease (P_1_=0.001; P_2_=0.001; P_3_<0.001) and tumor grade (P_1_=0.003; P_2_=0.015; P_3_=0.001).

### Correlation of IAP expression levels with bladder cancer prognosis

**Figure 3:**
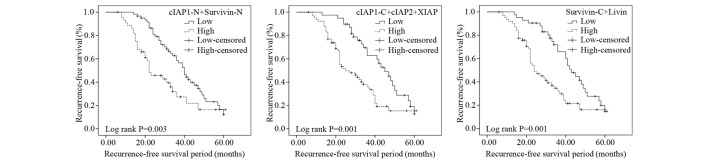
Kaplan-Meier estimates of recurrence-free survival according to the expression levels of each group of IAP family members. Significant differences are observed among the high and low combinative expression subgroups, which suggests the predictive value of the combination of IAP expression levels for bladder cancer. Log-rank test P-values are listed for each parameter. Low_cIAP1-N+Survivin-N_=scoring 0 and +1–2; Low_cIAP1-C+cIAP2+XIAP_=scoring 0 and +1–3; Low_Survivin-C+Livin_=scoring 0 and +1–2; High_cIAP1-N+Survivin-N_=scoring +3–4 and +5–6; High_cIAP1-C+cIAP2+XIAP_=scoring +4–6 and +7–9; High_Survivin-C+Livin=scoring_ +3–4 and +5–6.

Correlation between the expression parameters of each group of IAPs and tumor recurrence-free survival times were evaluated by Kaplan-Meier survival analysis with a log-rank statistic to determine significance. As demonstrated in Fig. 3, the mean recurrence-free survival times were significantly decreased in the high IAP expression groups compared with the low IAP expression groups (lowcIAP1-N+Survivin-N: 36.4 months, high_cIAP1-N+Survivin-N_: 24.4 months, P=0.003; low_cIAP1-C+cIAP2+XIAP_: 39.2 months, high_cIAP1-C+cIAP2+XIAP_: 26.6 months, P=0.001; and low_Survivin-C+Livin_: 39.4 months, high_Survivin-C+Livin_: 25.8 months, P=0.001). In a multivariate analysis based on a Cox proportional hazards regression model, we tested the independent predictive value of the expression of each group of IAPs, and the relevant clinical and pathological parameters, including gender, age, number of tumors, the presence of muscle-invasive disease and tumor grade. As demonstrated in Table III, the expression of each IAP group was a significant independent prognostic marker for recurrence-free survival (P_cIAP1-N+Survivin-N_=0.016; P_cIAP1-C+cIAP2+XIAP_=0.029; P_Survivin-C+Livin_=0.032), in addition to the presence of muscle-invasive disease and high tumor grade.

### Correlation between cIAP1-N expression and Survivin-N expression

**Figure 4:**
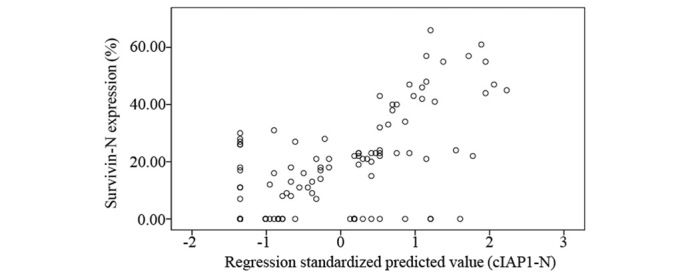
Linear regression plot between cIAP1-N expression and Survivin-N expression. Spearman’s analysis: r=0.55, P<0.001 (SPSS 13.0). cIAP1-N, nuclear cIAP1; Survivin-N, nuclear Survivin.

To investigate whether cIAP1-N expression was correlated with Survivin-N expression in bladder cancer patients, a Spearman’s correlation analysis was used. The results revealed a significant positive correlation between cIAP1-N expression and Survivin-N expression (r=0.55, P<0.001) in bladder cancer (Fig. 4).

## Discussion

The transformation of normal cells into carcinoma can be regarded as a powerful alteration of cell fate. Apoptotic pathways that should be tightly controlled are so significantly perturbed by carcinogenic factors that the cell fate is altered. In recent decades, complex networks of apoptotic and antiapoptotic proteins that govern apoptosis have been found to be biomarkers of disease or potential therapeutic targets against cancer, and are receiving increasing attention from academics and clinicians.

In the present study, we detected the overall expression trends for Survivin, cIAP1, cIAP2, XIAP and Livin in normal bladder tissues and bladder cancer tissues through western blot analysis. We then used immunohistochemical analysis to determine the localization and expression levels of each protein in low- and high-grade bladder cancer tissues. We demonstrated that Livin and Survivin were not detected in normal bladder tissues, while immunoreactivity for the other antibodies was found at the corresponding molecular weights in both normal and cancerous tissues. Subsequently, the immunohistochemical analysis data revealed that cIAP1 and Survivin exhibited a marked tendency toward nuclear expression in the high-grade bladder cancer panel compared with the low-grade panel. Based on these findings, we classified the five IAP members into three groups (1, cIAP1-N+Survivin-N; 2, cIAP1-C+cIAP2+XIAP; and 3, Survivin-C+Livin) and analyzed their corresponding clinical and pathological parameters in bladder cancer.

In IAPs, cIAP1, which is identified from tumor necrosis factor receptor (TRAF2)-related proteins, is a nuclear protein and it translocates to the cytosol in response to apoptotic signals that activate caspases. The nuclear localization of cIAP1 is dependent on its BIR domains. cIAP1 regulates the cell cycle, and its overexpression causes genomic alterations due to defects in cell division, possibly through interference with Survivin (20). Survivin, the smallest IAP member, contains a single BIR domain and is found in both the cytoplasm and nuclei of cells. Nuclear Survivin was suspected to control cell division, whereas cytoplasmic/mitochondrial Survivin was considered to be cytoprotective (21). The nuclear localization mechanisms of cIAP1 and Survivin are similar: i)cIAP1 and Survivin are nuclear shuttling proteins whose subcellular localization is mediated by the CRM1-dependent nuclear export pathway; ii) cIAP1 and Survivin both contain CRM1-dependent leucine-rich nuclear export signals (NES) to regulate nuclear export; and iii) the CRM1-mediated export of cIAP1 and Survivin may be inhibited by leptomycin B (LMB), which leads to the accumulation of cIAP1 and Survivin in the nuclei of cancer cells. Furthermore, Survivin and cIAP1 both localize to mid-body microtubules at telophase and interact with each other during mitosis. Therefore, the combination of cIAP1-N and Survivin-N may be an important factor in cell apoptosis and proliferation.

XIAP has been identified as one of the most potent inhibitors of caspases and apoptosis (22). As members of the IAP family, XIAP is structurally similar to cIAP1and cIAP2. These IAPs contain three baculovirus IAP repeats (BIR1, BIR2 and BIR3) and a C-terminal RING domain. Jin *et al* demonstrated that cIAP1, cIAP2 and XIAP were able to act cooperatively via non-redundant pathways to regulate genotoxic stress-induced nuclear factor-κB activation (16). Gill *et al* reported that the simultaneous knockdown of XIAP, cIAP1 and cIAP2 made prostate cancer cells susceptible to apoptosis, thus decreasing cell survival and proliferation (23). These observations suggest that the expression levels of cIAP1, cIAP2 and XIAP in cancer cells may be involved in determining the equilibrium between proliferation and apoptosis. cIAP1, cIAP2 and XIAP are not only found in cancer cells, but are also broadly expressed at the mRNA level in normal cells (24). It has been demonstrated that IAPs in normal tissues may have several potential physiological roles, including regulation of the immune system, the response to cell damage, and cell survival and differentiation.

Livin, which is also referred to as melanoma inhibitor of apoptosis protein (ML-IAP) or kidney inhibitor of apoptosis protein (KIAP), is the most recently identified member of the IAP family. Its anti-apoptotic mechanism is mediated through the inhibition of caspase-3, -7 and -9, and by its E3 ubiquitin-ligase-like activity, which promotes the degradation of Smac/DIABLO (25). One of our previous studies reported that Livin regulated prostate cancer cell invasion by impacting the NF-κB signaling pathway, and the expression of FN and CXCR4 (26). Livin and Survivin have been demonstrated to be extensively expressed in many types of cancer and either not expressed or expressed at substantially lower levels in their normal tissue counterparts. Xi *et al* demonstrated that high expression levels of both Survivin and Livin may influence the prognosis of human colorectal cancer (27). Li *et al* demonstrated that Livin and Survivin may be involved in the pathogenesis and progression of adult patients with acute lymphoblastic leukemia (28). The interpretation of these data suggested that the exclusively cancer cell-expressed Livin and Survivin may be valuable biomarkers of different types of cancer.

The Mann-Whitney U test analysis in the present study revealed that the expression of each IAP group was significantly correlated with the presence of muscle-invasive disease and tumor grade. The data for the individual IAPs demonstrated that only the expression levels of cIAP1-N and Survivin-N were correlated with tumor stage and grade, while the expression levels of cIAP1-C, cIAP2, XIAP, Survivin-C and Livin were not correlated with tumor stage or grade (data not shown). The results of the single IAPs were in agreement with previous studies (11–14). The discrepancies between the individual data and the combined data demonstrate that the functionally similar expression levels of multiple IAPs may be a more valuable biomarker of bladder cancer development and progression than the expression levels of individual IAPs. The Kaplan-Meier survival analysis with a log-rank statistic suggested that each classified group was correlated with bladder cancer recurrence. Our multivariate analysis demonstrated that the expression of each classified group had a prognostic effect that was independent of tumor stage and grade. Furthermore, the Spearman’s analysis revealed a significant positive correlation between cIAP1-N and Survivin-N expression in bladder cancer patients. This result suggests that the suppression of either cIAP1 or Survivin expression affected both proteins and could contribute to the development of a gene therapy-based treatment for bladder cancer. However, the findings from the multivariate analyses in our study are based on a limited number of cases and clinicopathological parameters, and require confirmation from analyses of larger cohorts.

Recently, due to the unique pathological overexpression of IAPs that has been documented in cancer tissues and the development of translational medicine approaches to therapy, a novel and promising strategy has been to develop targeted therapies against IAPs for the treatment of malignancy. Several small molecules, such as deguelin and D,L-sulforaphane, have been shown to downregulate IAPs, release their inhibitory activity, reduce toxicity and improve the efficacy of cancer treatments (29,30). In the present study, we examined the overall trends of IAP expression and compared the selected expression levels of multiple IAPs with corresponding clinical and pathological tumor features, and with prognosis, in bladder cancer patients. Our data may indicate a more valuable multi-gene therapeutic target for the development of effective inhibitors and may be the theoretical basis of a translational medicine approach to bladder cancer gene therapy.

The molecular mechanisms of bladder cancer development and progression are complicated, and are likely to involve the interaction of tumor suppressor genes, oncogenes, growth factors, adhesion molecules and angiogenic factors that together induce a normal transitional cell to acquire a malignant phenotype. Thus, further research is required to improve our understanding of the mechanisms and pathways of bladder cancer, in order to clinically alter the diagnosis and treatment of bladder cancer patients.

## Figures and Tables

**Table I:** Expression of IAP family members in bladder cancer and normal bladder urothelium.

Variable	n	Survivin		cIAP1		cIAP2		XIAP		Livin	
		PC (%)	P-value	PC (%)	P-value	PC (%)	P-value	PC (%)	P-value	PC (%)	P-value
BCC	105	74 (70)	<**0.001**	89 (85)	**0.001**	91 (87)	**<0.001**	69 (66)	**0.002**	41 (39)	**<0.001**
NBU	36	0 (0)		21 (58)		20 (56)		13 (36)		0 (0)	

IAP, inhibitors of apoptosis; BCC, bladder cancer cell; NBU, normal bladder urothelium; PC, patient cases. Significantly different values are indicated in bold.

**Table II:** Correlation between clinicopathological parameters and the expression level of each IAP group. cIAP1-N+Survivin-N

Variable	n	cIAP1-N+Survivin-N				P-value	cIAP1-C+cIAP2+XIAP				P-value	Survivin-C+Livin				P-value
		0	+1	+2	+3		0	+1	+2	+3		0	+1	+2	+3	
Gender																
Male	75	7	36	24	8	0.941	1	28	46	0	0.356	17	12	43	3	0.839
Female	30	1	16	12	1		0	10	18	2		4	9	17	0	
Age																
<Mean	55	3	28	19	5	0.669	0	22	32	1	0.59	10	13	32	0	0.62
Mean	50	5	24	17	4		1	16	32	1		11	8	28	3	
Number																
Single	72	8	32	26	6	0.827	1	29	41	1	0.135	15	13	41	3	0.702
Multiple	33	0	20	10	3		0	9	23	1		6	8	19	0	
Stage^a^																
NMIBC	60	6	36	17	1	**0.001**	0	31	28	1	**0.001**	19	13	28	0	<**0.001**
MIBC	45	2	16	19	8		1	7	36	1		2	8	32	3	
Grade^b^																
Low	70	7	39	22	2	**0.003**	0	32	37	1	**0.015**	20	14	36	0	**0.001**
High	35	1	13	14	7		1	6	27	1		1	7	24	3	

cIAP1-N, nuclear cIAP1; Survivin-N, nuclear Survivin; cIAP1-C, cytoplasmic cIAP1; Survivin-C, cytoplasmic Survivin; NMIBC, non-muscle-invasive bladder cancer; MIBC, muscle-invasive bladder cancer. Group _cIAP1-N+ Survivin-N_: 0=0; +1=+1–2; +2=+3–4; +3=+5–6; Group _cIAP1-C+ cIAP2+XIAP_: 0=0; +1=+1–3; +2=+4–6; +3=+7–9; Group _Survivin-C + Livin: 0_=0; +1=+1–2; +2=+3–4; +3=+5–6.

a Tumor stage was determined using the 2002 TNM classification system.

b Tumor grade was determined using the 2004 WHO grading system. Significantly different values are indicated in bold.

**Table III:** Cox regression analysis of prognostic parameters for recurrence-free survival in bladder cancer (Backward: LR).

Variable	B	SE	Wald	df	P-value	Exp (B)	95% CI for Exp (B)	
							Lower	Upper
cIAP1-N+Survivin-N	0.617	0.256	5.827	1	**0.016**	0.539	0.327	0.89
Stage (MI vs. NMI)	0.959	0.364	6.962	1	**0.008**	0.383	0.188	0.781
Grade (high vs. low)	0.752	0.353	4.55	1	**0.033**	0.471	0.236	0.941
cIAP1-C+ cIAP2+XIAP	0.585	0.267	4.792	1	**0.029**	0.557	0.33	0.941
Stage (MI vs. NMI)	0.937	0.376	6.195	1	**0.013**	0.392	0.187	0.82
Grade (high vs. low)	0.653	0.355	3.374	1	0.066	0.521	0.259	1.045
Survivin-C+Livin	0.575	0.268	4.59	1	**0.032**	0.563	0.332	0.952
Stage (MI vs. NMI)	0.942	0.378	6.221	1	**0.013**	0.39	0.186	0.817
Grade (high vs. low)	0.6	0.354	2.863	1	0.091	0.549	0.274	1.1

cIAP1-N, nuclear cIAP1; Survivin-N, nuclear Survivin; cIAP1-C, cytoplasmic cIAP1; Survivin-C, cytoplasmic Survivin; NMI, non-muscle invasive; MI, muscle invasive. Low_cIAP1-N+Survivin-N_=scoring 0 and +1–2; Low_cIAP1-C+cIAP2+XIAP_=scoring 0 and +1–3;Low_Survivin-C+Livin_=scoring 0 and +1–2; High_cIAP1-N+ Survivin-N_=scoring +3–4 and +5–6; High_cIAP1-C+cIAP2+XIAP_=scoring +4–6 and +7–9; High_Survivin-C+Livin_=scoring+3–4 and +5–6. B, coefficient; SE, standard error; df, degrees of freedom. Significantly different values are indicated in bold.
